# 

**Published:** 1900-06-02

**Authors:** 


**CADBURY'S COCOA** ABSOLUTELY PURE.

### THE HOSPITAL

**CADBURY'S COCOA** NO DRUGS OR ALKALIES USED.

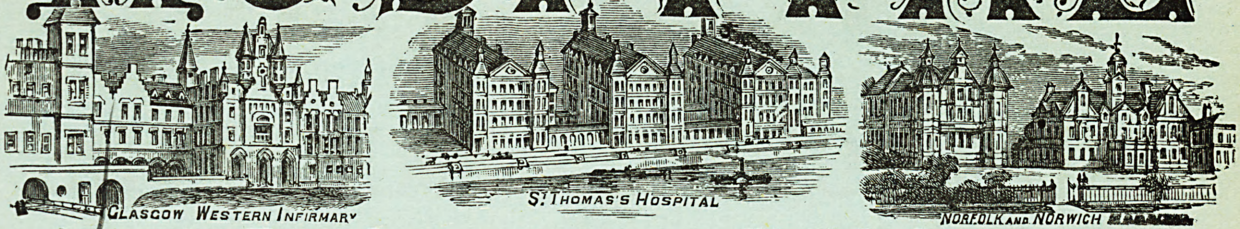

No. 714. Vol. XXVIII. [Registered as a Newspaper.] Saturday, June 2, 1900. [Subscription Terms, see p. 148.] **PRICE TWOPENCE.**

